# Xanthohumol Impairs the PMA-Driven Invasive Behaviour of Lung Cancer Cell Line A549 and Exerts Anti-EMT Action

**DOI:** 10.3390/cells10061484

**Published:** 2021-06-12

**Authors:** Adrianna Sławińska-Brych, Magdalena Mizerska-Kowalska, Sylwia Katarzyna Król, Andrzej Stepulak, Barbara Zdzisińska

**Affiliations:** 1 Department of Cell Biology, Faculty of Biology and Biotechnology, Institute of Biological Sciences, Maria Curie-Sklodowska University, Akademicka 19, 20-033 Lublin, Poland; 2 Department of Virology and Immunology, Faculty of Biology and Biotechnology, Institute of Biological Sciences, Maria Curie-Sklodowska University, Akademicka 19, 20-033 Lublin, Poland; magdalena.mizerska-dudka@poczta.umcs.lublin.pl (M.M.-K.); basiaz@poczta.umcs.lublin.pl (B.Z.); 3 Laboratory of Neuro-oncology, Mossakowski Medical Research Institute, Polish Academy of Sciences, Pawinskiego 5, 02-106 Warsaw, Poland; skrol@imdik.pan.pl; 4 Department of Biochemistry and Molecular Biology, Faculty of Medicine, Medical University of Lublin, Chodzki 1, 20-093 Lublin, Poland; andrzej.stepulak@umlub.pl

**Keywords:** xanthohumol, lung cancer, migration, invasion, MMPs, EMT, FAK, AKT, ERK

## Abstract

Xanthohumol (XN), the main prenylated flavonoid from hop cones, has been recently reported to exert significant proapoptotic, anti-proliferative, and growth inhibitory effects against lung cancer in both in vitro and in vivo studies. However, its anti-metastatic potential towards this malignancy is still unrevealed. Previously, we indicated that the human lung adenocarcinoma A549 cell line was sensitive to XN treatment. Therefore, using the same tumour cell model, we have studied the influence of XN on the phorbol-12-myristate-13-acetate (PMA)-induced cell migration and invasion. The effects of XN on the expression/activity of pro-invasive MMP-9 and MMP-2 and the expression of MMP inhibitors, i.e., TIMP-1 and TIMP-2 (anti-angiogenic factors), were evaluated. Additionally, the influence of XN on the production of the key pro-angiogenic cytokine, i.e., VEGF, and the release of TGF-β, which is both a pro-angiogenic cytokine and an epithelial-mesenchymal transition (EMT) stimulator, was studied. Furthermore, the influence of XN on the expression of EMT-associated proteins such as E-cadherin and α-E-catenin (epithelial markers), vimentin and N-cadherin (mesenchymal markers), and Snail-1 (transcriptional repressor of E-cadherin) was studied. To elucidate the molecular mechanism underpinning the XN-mediated inhibition of metastatic progression in PMA-activated cells, the phosphorylation levels of AKT, FAK, and ERK1/2 kinases, which are signalling molecules involved in EMT program activation, were assayed. The results showed that XN in non-cytotoxic concentrations impaired the PMA-driven migratory and invasive capacity of A549 cells by decreasing the level of expression of MMP-9 and concomitantly increasing the expression of the TIMP-1 protein, i.e., a specific blocker of pro-MMP-9 activation. Moreover, XN decreased the PMA-induced production of VEGF and TGF-β. Furthermore, the XN-treatment counteracted the PMA-induced EMT of the A549 cells by the upregulation of E-cadherin and α-E-catenin and the downregulation of N-cadherin, vimentin, and Snail-1 expression. The proposed mechanism underlying the anti-invasive XN activity involved the inhibition of the ERK/MAPK pathway and suppression of FAK and PI3/AKT signalling. Our results suggesting migrastatic properties of XN against lung cancer cells require further verification in in vivo assays.

## 1. Introduction

Lung cancer (LC) is the leading cause of cancer-related mortality among males and females in all industrialized countries [1]. According to the Global Cancer Observatory (GCO), approximately 11.6% of total new cancer cases (2.1 million) and 18.4% (1.8 million) of total cancer-related deaths were associated with lung cancer in 2018 [2]. Based on different morphological features, lung cancer is categorized into small-cell lung cancer (SCLC; accounting for 15% of LC patients) and non-small-cell lung cancer (NSCLC; 85% of LC patients). The group of NSCLC comprises lung adenocarcinoma (40%), lung squamous cell carcinoma (30%), and large cell lung cancer (15%) [3]. Despite the recent advances in early detection and management of lung cancer therapy, the survival rates for LC patients have only marginally improved. In addition to the systemic drug toxicity and multidrug resistance during chemotherapy, aggressive local invasion and metastases are the major factors in the unfavourable outcome of lung cancer patients [4]. Therefore, the inhibition of invasion and metastasis development is considered one of the most important goals in the treatment of patients with advanced lung cancer disease [5].

In the last few decades, the worldwide interest in phytotherapy used for the prevention or treatment of several chronic diseases has increased considerably [6]. Compared with synthetic drugs, most phytotherapeutics are safer and produce fewer side effects, hence their use is commonly accepted [7]. Hop plants are emerging as one of the most promising sources of numerous bioactive compounds. Due to their diverse biological activities, including antimicrobial, antifungal, antioxidant, anti-inflammatory, anti-genotoxic, and anti-cancer properties, they are applied in medicine and industry [8,9]. Hop-derived constituents, in particular xanthohumol (XN, a prenylchalcone), display strong anticancer and chemopreventive potential [9]. Our research and previous reports have documented the antiproliferative and proapoptotic activities of XN against larynx cancer [10], lung cancer [11,12], multiple myeloma [13], prostate cancer [14,15], ovarian and breast cancer [16], hepatoma [17], gliomas, and leukaemia [18,19]. The key cellular and molecular mechanisms of the XN action involve the induction of cell cycle arrest in the G0/G1 or S phase, regulation of apoptosis-associated targets (Bax, p53, procaspase-8, -9, -3, Bcl-2, Bcl-xL, survivin, cIAP-1, cIAP-2, XIAP), the inhibition of phosphatidyl-inositol-3-(PI3)-kinase and/or mitogen-activated protein kinase (MAPK) signalling pathways, and the modulation of the activity of NF-κB and STAT-3 transcription factors, which are frequently expressed constitutively in a majority of malignant cells [9,20]. Furthermore, XN has been demonstrated to enhance the susceptibility of tumour cells to radio- and chemotherapy and overcome drug-resistance [20]. In a xenograft murine model, XN was reported to restrict the significantly distant dissemination of highly aggressive L1210 lymphocytic leukaemia cells to CNS and prolong animal survival [21]. It also impaired the metastatic capability of a breast cancer cell line by decreasing its motility and repressing the expression of matrix metalloproteinases (MMPs) [22]. However, the ability of XN to suppress the metastatic potential of lung tumour cells has to be elucidated.

Metastatic progression is a precisely controlled multi-step cell-biological process involving specific cell-to-cell and cell-to-ECM (extracellular matrix) interactions, as well as cell movement and invasion in the surrounding microenvironment, which results in the entry of cancer cells into the vasculature and spread to distant organs. The metastasis of epithelial tumours is facilitated by the transitioning of cancer cells from a non-motile epithelial phenotype into a migratory mesenchymal-like phenotype, a phenomenon known as epithelial to mesenchymal transition (EMT) [23]. The hallmark of the EMT process is the loss of the expression of epithelial markers and concomitant upregulation of mesenchymal markers, as well as the increased activity of MMPs (zinc-dependent endopeptidases), including MMP-2 and MMP-9, which is associated with the invasive phenotype [24,25]. E(epithelial)-cadherin (a calcium-dependent cell adhesion glycoprotein) and α-E-catenin (a cytoplasmic molecule) are epithelial markers and the principal components of adherence junctions with an invaluable role in intercellular connectivity, tissue integrity, and cell polarity. They play a migration-suppressive role in tumours [26,27]. In contrast, the expression of N(neural)-cadherin in cancer cells following E-cadherin loss (so called “cadherin switch”) is associated with tumour progression and increased migratory and the invasive behaviour of cancer cells [23,28]. Additionally, the stromal cell-specific intermediate filament vimentin is characterized by oncogene properties, promoting faster tumour growth, cell migration, and invasion [29].

Another group of proteins, i.e., MMPs, contribute to tumour invasion by degrading and remodelling ECM and basement membrane components and promoting neoangiogenesis via increased secretion of the key pro-angiogenic cytokine, i.e., the vascular endothelial growth factor (VEGF), from cancer cells [30,31]. Of all human MMPs described so far, gelatinases (or type IV collagenases), i.e., MMP-2 (gelatinase-A) and MMP-9 (gelatinase-B), are recognized to be apparently correlated with pathological grading, staging, metastasis, and poor prognosis in patients with lung adenocarcinoma [31,32]. The activity of MMPs is tightly controlled by, e.g., the presence of endogenous tissue inhibitors TIMPs, i.e., multifunctional proteins known as anti-tumorigenic and anti-angiogenic factors [33]. Among TIMPs, TIMP-1 preferentially inhibits MMP-9, while TIMP-2 is a more effective inhibitor of MMP-2 [24,33]. 

It has been demonstrated that the tumour-produced cytokine, i.e., the transforming growth factor (TGF-β), is an inducer of the EMT process. TGF-β can work as a proto-oncogene or a tumour suppressor, depending on the cell context and tumorigenesis stage [34]. Indeed, in the late phase of tumour development, this cytokine has been shown to exhibit pro-cancerous effects attributable to tumour growth acceleration, the stimulation of cellular dedifferentiation and neoangiogenesis, and the suppression of apoptosis and the immune system. TGF-β signalling can induce the expression of the transcription factor Snail-1, which in turn represses the transcription of E-cadherin and thus promotes EMT [28]. Moreover, TGF-β upregulates N-cadherin expression in NSCLC [35].

Among the many identified regulators of EMT (e.g., Snail, ZEB, Twist), Snail-1 has emerged to be the most powerful repressor of the E-cadherin gene. It also augments the expressions of vimentin, fibronectin, and “stemness” genes, as well as the mRNA levels of MMP-2 and MMP-9 [25,36]. 

Both phosphoinositide-3 kinase/protein kinase B (PI3K/AKT) and Ras/MEK/ERK (extracellular signal-regulated protein kinase) cascades have been found to be connected with TGF-β-induced EMT and ERK1/2, and AKT has been recognized to provoke the EMT-like phenotype via the repression of Snail-1 and/or ZEB-1 transcription factors [37,38]. Moreover, AKT and ERK activation is known to serve not only cell proliferation and survival, but also angiogenesis and tumour metastasis via regulation of MMPs and VEGF genes [39,40]. In turn, focal adhesion kinase (FAK) phosphorylation was reported to activate a multitude of protein kinases, such as ERK and PI3, contributing to the improvement of the proliferative, migratory, and invasive ability of different types of cancer cells [41,42].

Tumour metastasis can be promoted and enhanced via multiple mechanisms. Phorbol 12-myristate 13-acetate (PMA) is a known selective activator of protein kinase C commonly used to induce an invasive and metastatic phenotype in in vitro cultured cancer cells [43,44]. Noteworthy, PMA has recently been evidenced to magnify the motility and aggressiveness of the weakly invasive A549 lung adenocarcinoma cell line by inducing MMPs and the epithelial-mesenchymal transition (EMT) process [44,45,46]. Here, we provided evidence that XN inhibits the PMA-induced migration and invasion of A549 cells. These effects were associated with the TIMP-1-dependent downregulation of MMP-9 activity and modulation of VEGF, TGF-β and EMT-related protein expression, as well as the AKT, FAK, and ERK1/2 signalling pathways.

## 2. Materials and Methods

### 2.1. Chemicals and Reagents

Xanthohumol (XN) was purchased from Cayman Chemical (Ann Arbor, MI, USA). Phenylmethylsulfonyl fluoride (PMSF), Phorbol-12-myristate-13-acetate (PMA), dimethyl sulfoxide (DMSO), fetal bovine serum (FBS), culture media, penicillin-streptomycin solution, L-glutamine, LY294002 (a PI3 kinase inhibitor), Y15 (a FAK kinase inhibitor), protease and phosphatase inhibitor cocktail, MTT, and the BCA Protein Assay Kit were obtained from Sigma-Aldrich Chemicals (St. Louis, MO, USA). SC772984 (an ERK1/2 inhibitor) was supplied by Selleck Chemicals LLC, Houston, TX, USA. Triton X-100, Tris, SDS, precision plus protein TM standards, polyvinylidene difluoride (PVDF) membranes, and the ECL plus Western blotting detection kit were obtained from Bio-Rad Laboratories (Hercules, CA, USA). Primary antibodies against MMP-9, TIMP-1, TIMP-2, and N-cadherin were purchased from Santa Cruz Biotechnology (Santa Cruz Biotechnology Inc. Dallas, TX, USA) and those against MMP-2 were provided by Novus Biological (Littleton, CO, USA). Primary antibodies against vimentin, α-(E)-catenin, E-cadherin, and all secondary antibodies conjugated with horseradish-peroxidase or FITC were purchased from Cell Signaling Technology (Danvers, MA, USA). Other reagents used were of analytical grade and were obtained from commercial sources. The stock solutions of XN were prepared in DMSO and further diluted in culture medium. The final concentration of DMSO was lower than 0.1%. To exclude any toxic effects of DMSO, the 0.1% concentration of solvent was used as a blank reagent.

### 2.2. Cell Culture

The human Caucasian non-small-cell lung carcinoma A549 line was purchased from the American Type Cell Culture Collection (Manassas, VA, USA). The A549 adenocarcinoma cells were grown in a 2:1 mixture of Dulbecco’s modified Eagle medium and Ham’s F12 medium supplemented with 10% fetal bovine serum, a 1% penicillin-streptomycin solution, and 2 mM L-glutamine. The cells were maintained in an incubator with a humidified atmosphere of 95% air and 5% CO_2_ in air at 37 °C.

### 2.3. Assessment of Cell Viability

The A549 cells (1 × 10^4^ cells/well) in a complete medium were seeded into 96-well microplates (Nunc, Roskilde, Denmark). The next day, the medium was removed and some cells were left untreated, whereas others were treated with PMA at a concentration of 50 nM (prepared in medium with 1% FBS) or exposed to combinations of 50 nM PMA with different concentrations of XN (2.5–80 μM). After 24 h, cell viability was measured by the MTT (3-(4,5-dimethylthiazol-2-yl)-2,5-diphenyltetrazolium bromide) assay, as described previously [11]. Changes in the viability of the A549 cells were calculated as a percentage of the PMA-stimulated XN-untreated group, which was considered to be 100% alive and was compared to each PMA-stimulated XN-treated group. 

### 2.4. Wound Healing Assay

The A549 cells (5 × 10^5^ cells/mL) were seeded in culture dishes (4 cm in diameter, Nunc) in a complete medium for 24 h. Then, the plates were scratched with micropipette tips to create one linear wound (with constant width). After removing the cellular debris with PBS, the cells were pre-treated for 4 h with 1% FBS media containing various concentrations of XN (0–10 μM). Following further 24-h co-incubation in the presence of PMA, the dishes were stained according to the May–Grünwald–Giemsa method and then observed under an Olympus BX51 System Microscope (Olympus Optical, Tokyo, Japan). Cells that migrated to the wound areas were counted on micrographs and the results were taken as the mean number of cells that had migrated to 50 selected fields taken from four micrographs. The changes in the A549 cell migration were expressed as % of the PMA-stimulated XN-untreated control group level (100%).

### 2.5. Transwell Invasion Assay

The in vitro invasive potential of lung cancer cells was studied in 24-well transwell chambers with a polycarbonate membrane (8.0 μm pore size) coated with ECMatrixTM, a reconstituted basement membrane matrix of proteins derived from the Engelbreth Holm-Swarm mouse tumour (Merck Millipore Corporation, Burlington, MA, USA), according to the manufacturer’s instructions. The bottom transwell chambers were filled with 500 µL of complete media with 10% FBS. The top chambers were seeded with A549 cells (1 × 10^5^ cells/well) suspended in 250 µL of serum-free medium containing the selected concentrations of XN (0, 2.5, 5, or 10 µM). In the experiment with the inhibitors, the cells were also exposed to XN (5 µM) in combination with Y15 (a FAK inhibitor, 5 µM), LY294002 (a PI3/AKT inhibitor, 10 µM), SCH772984 (an ERK inhibitor, 5 µM), or with each of them separately. After 4-h pre-incubation, the cells were co-stimulated with PMA (50 nM) for 24 h to allow invasion through the membrane. At the end of the treatment, the invaded cells were dissociated from the lower surface of the insert membrane using the cell detachment buffer, and subsequently lysed and labelled with fluorescence CyQuant GR dye (which binds to cellular nucleic acids). The fluorescence intensity was measured at wavelengths of 480/520 nm (Ex/Em) on a bottom-reading fluorescence plate reader (Victor X4, Bioanalytic, PerkinElmer, Turku, Finland).

### 2.6. Immunofluorescence Analysis

For the fluorescence immunohistochemical evaluation of protein expression, the cells were cultured up to 70% confluence in a 4-well chamber slide (Nunc Lab-Tek Chamber Slide Systems, Thermo Fisher Scientific Inc., Waltham, MA, USA). Next day, the culture medium was removed, and the cells were pre-treated with different concentrations of XN (prepared in a medium with 1% of FBS) for 4 h and then co-treated with PMA (50 nM) for 24 h. Then, the cells were fixed with 4% paraformaldehyde (Sigma-Aldrich) in PBS for 15 min, permeabilized with 0.5% Triton X-100 (Sigma-Aldrich) for 15 min, and further blocked with blocking buffer (1% BSA, 0.25% Triton X-100 in PBS, pH 7.4) for 1 h. Incubation with primary rabbit anti-vimentin and anti-E-cadherin (diluted 1:250) was conducted overnight at 4 °C. After washing, the cells were incubated with goat anti-rabbit FITC-conjugated secondary antibody (diluted 1:500) for 1 h at room temperature. After washing with PBS, the cells were viewed using an Olympus BX51 System Microscope.

### 2.7. Gelatine Zymography

The A549 cells were seeded in 24-well plates at a density of 5 × 10^5^ cell/well and cultured overnight. The next day, the cells were pre-treated with various concentrations of XN (0, 2.5, 5, or 10 µM) for 4 h and then co-stimulated with PMA for another 24 h. After the treatment, the conditioned media were collected, centrifuged to remove debris, and stored at −20 °C until use. The protein content was determined using the BCA kit. Samples with equal protein concentrations were mixed with nonreducing 4 × Laemmli buffer and subjected to electrophoresis in 10% SDS-PAGE containing 0.1% (*w/v*) gelatine (Sigma-Aldrich). The gel was washed with 2.5% Triton X-100 (*v/v*) at room temperature for 30 min to remove SDS and allow the protein to renature; subsequently, it was incubated in substrate buffer (50 mM Tris-HCl, pH 7.5, 1 mM ZnCl_2_, and 5 mM CaCl_2_) at 37 °C for 24 h. The gel was then stained with 0.5% (*w/v*) Coomassie blue (Sigma-Aldrich) in 10% acetic acid (*v/v*) and 30% methanol (*v/v*) and then de-stained with the same solution without Coomassie blue. Gelatinolytic activities were detected as unstained bands against the background of Coomassie blue-stained gelatine. Importantly, in such SDS-containing gel, the latent form of MMP-9, i.e., pro-MMP-9, and the activated gelatinase exhibit gelatinolytic activity. Therefore, the word ‘‘activity’’ was used to indicate the total gelatinolytic activity measured in the conditioned media.

### 2.8. Western Blotting

The expression of MMPs, α-E-catenin, E-cadherin, N-cadherin, vimentin, and TIMPs was determined using Western blot as previously described [11]. Briefly, the A549 cells were grown in 10-cm diameter plastic plates to 80% confluence. After 4-h pre-treatment with XN (0–10 µM) in a medium containing 1% FBS, the cells underwent 24-h co-incubation with PMA (50 nM). Subsequently, the cells were harvested, lysed on ice in RIPA buffer supplemented with protease inhibitor cocktail, and centrifuged (14,000 rpm for 10 min at 4 °C). The total protein concentrations were determined using a BCA Protein Assay Kit. Samples with equal protein concentrations were electrophoresed, electrotransferred to polyvinylidene difluoride (PVDF) membranes, and immunoblotted. Specific signals on blotted membranes were detected with the ECL plus Western blotting detection kit. Chemiluminescence was visualized using ChemiDoc + XRS (Bio-Rad Laboratories Inc., Hercules, CA, USA). The immunoblot bands were quantified densitometrically by ImageJ and normalized to β-actin. The effect of XN on the protein levels was given as the relative expression percentage compared to the PMA-stimulated non-treated control group, which was established as 100%.

### 2.9. Assay of MMPs, TIMPs, VEGF, and TGF-β Production

The amounts of MMP-2, MMP-9, TIMP-1, TIMP-2, VEGF, and TGF-β present in the culture media of the A549 cells were estimated using Bioassay Biotechnology Laboratory ELISA kits (Shanghai, China) according to the manufacturer’s instructions. The reaction products were quantified spectrophotometrically by measuring absorbance at 450 nm using an E-max Microplate Reader (Molecular Devices Corporation, Menlo Park, CA, USA). The Concentrations of these molecules were calculated based on the standard curve drawn, with known concentrations of recombinant proteins and represented graphically. Briefly, the A549 cells were seeded in 24-well plates at a density of 5 × 10^5^ cell/well and cultured overnight. Next, the cells were treated with the indicated concentrations of XN (prepared in medium containing 1% FBS) for 4 h and then co-treated with PMA (50 nM) for 24 h. In some experiments, prior to the 24-h PMA stimulation, the cells were pre-treated with Ly294002 (10 µM), Y15 (5 µM), or SCH772984 (5 µM), without or in combination with XN (5 µM). At the end of all treatments, the supernatants were collected, centrifuged to remove debris, and stored at −20 °C until use.

### 2.10. Determination of the FAK, AKT, and ERK1/2 Activity Status and Snail-1 Transcription Factor Expression

The A549 cells were seeded into 10-cm diameter plastic plates and cultured in the growth medium to 80% confluence. Then, the medium was replaced with fresh medium containing 1% FBS with the indicated concentrations of XN. After 4-h pre-treatment, the cells were co-incubated with PMA (50 nM) for 24 h. Subsequently, the media were removed and the cells were lysed in lysis buffer supplemented with PMSF and protease and phosphatase inhibitor cocktail according to the manufacturer’s protocol. The cell lysates were centrifuged at 14,000 rpm at 4 °C for 5 min and stored at −20 °C until analysis. Before the assay, the total protein concentrations in the cell lysates were determined with a BCA Protein Assay Kit, and samples containing equal amounts of total proteins were subjected to ELISA assays. The total FAK, phospho-AKT, and Snail-1 were determined using a commercial Human FAK ELISA kit, a Human phospho-AKT (Ser 473) ELISA kit, or a Human Snail Homolog 1 ELISA Kit, respectively (Bioassay Biotechnology Laboratory, Shanghai, China). Phospho-FAK was assessed with a Human phospho-FAK (Y397) ELISA Kit (Shanghai Coon Koon Biotech Co., Ltd., Shanghai, China). Total and phosphorylated ERK1/2 and total AKT were measured using the following PathScan^®^ ELISA kits: total p44/42 (ERK 1/2), phospho-p44/42 MAPK (Thr202/Tyr204), and total AKT Sandwich ELISA Kit (Cell Signaling Technology, Danvers, MA, USA). The ELISA tests were performed according to the manufacturer’s protocols, and the optical density was measured using an E-max Microplate Reader.

### 2.11. Statistical Analyses

Each experiment was repeated at least three times. Statistical analyses were conducted using GraphPAD Prism 5 (GraphPAD Software Inc. version 5.00., San Diego, CA, USA). The data were analysed by one-way ANOVA followed by Dunnett’s or Tukey’s multiple comparison tests. Values were represented as means ± SD, and *p* values < 0.05 were considered statistically significant.

## 3. Results

### 3.1. XN Inhibits PMA-Induced Migration and Invasion of Lung Adenocarcinoma Cells

**Figure 1:**
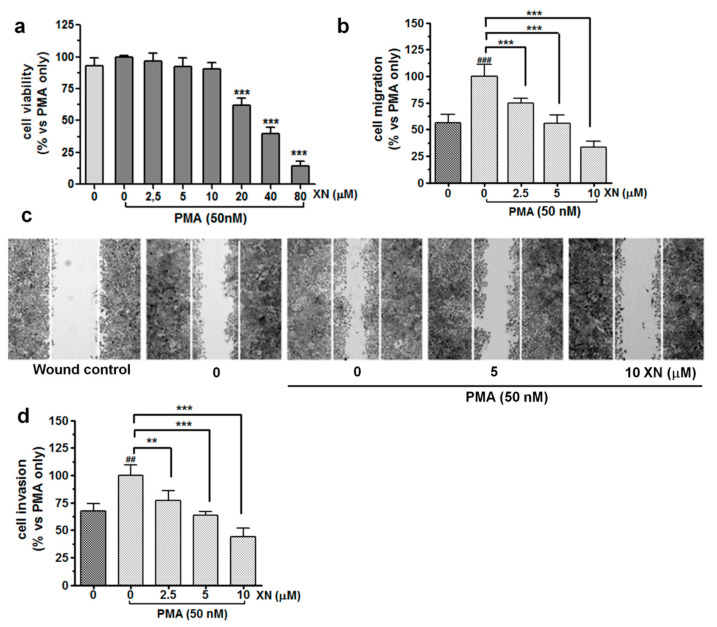
Impact of XN on the viability, migration, and invasion of PMA-induced A549 cells. (**a**) The effect of XN on the viability of PMA-induced A549 cells was determined with the MTT assay following 24-h exposure of these cells to a combination of different concentrations of XN (0–80 µM) with 50 nM PMA. The percentage of cell viability was calculated as a ratio of XN+PMA-treated cells to the PMA-only treatment. Data are expressed as the mean ± SD of three independent experiments, *** *p* < 0.001 in comparison to the PMA-only treated cells; one-way ANOVA test. (**b**) XN significantly decreases the PMA-activated cell migration in the wound healing assay. The bar graphs represent the number of migrated cells that were pre-treated for 4 h with the non-toxic concentrations of the compound before the 24-h co-incubation with PMA. Values are presented as the mean ± SD of three independent experiments, *** *p* < 0.001 in comparison to the PMA-only treatment; ### *p* < 0.001 in comparison to the non-stimulated cells; one-way ANOVA test. (**c**) Images of the migration (40-fold magnification) taken under a light microscope. (**d**) XN inhibits PMA-induced invasion of A549 cells in a concentration-dependent manner. After the treatment, the invasion of the cells was measured using a matrigel-based transwell invasion assay. The results are presented as the mean ± SD of three independent experiments, ** *p* < 0.01, *** *p* < 0.001 in comparison to the PMA-only treatment, ## *p* < 0.01 in comparison to the non-stimulated cells; one-way ANOVA test.

XN-mediated concentration-dependent inhibition of A549 cell viability was demonstrated previously [11]. In the present study, we determined the cytotoxic effect of the combined treatment of XN with PMA (50 nM) against the A549 cells after 24-h incubation by means of the MTT assay. As shown in Figure 1a, in the presence of PMA, XN at concentrations from 2.5 µM to 10 µM did not exhibit significant cytotoxicity.

A substantial reduction in the cell viability was observed after treatment with 20 µM or higher concentrations (40 and 80 µM) of XN. Additionally, the cytometric measurement of caspase-3 activity showed no pro-apoptotic action of XN used at concentrations up to 10 µM (Figure S1). Additionally, upon the treatment with 2.5, 5, or 10 µM, this compound did not inhibit DNA synthesis in the PMA-stimulated A549 cells (Figure S2). However, a clear anti-proliferative effect of XN was already seen at 20 µM. Therefore, non-cytotoxic and non-proliferative concentrations of XN, i.e., 2.5, 5, and 10 µM, were selected for further experiments.

The wound healing assay (Figure 1b,c) demonstrated that XN significantly inhibited the PMA-stimulated migration of the A549 cells in a concentration-dependent manner. After pre-treatment with 10 µM of XN, almost a 67% reduction in the number of motile cells was observed, compared with that in the PMA-only treatment (*p* < 0.001, Figure 1b). Similar data were found when the XN-pre-treated cells were allowed to migrate across the basement membrane extract (BME)-coated membrane. As shown in Figure 1d, the cell invasiveness stimulated by PMA (for 24 h) was significantly decreased by XN in a concentration-dependent manner. At the concentration of 10 µM, the invasion of the A549 cells was inhibited by approx. 56% in comparison to the PMA-treated control cells (*p* < 0.001). Taken together, these findings suggest that XN may play an important role in the inhibition of stimulated migration and invasion of lung cancer cells.

### 3.2. XN Decreased PMA-Induced Activity and Expression of MMP-9

**Figure 2:**
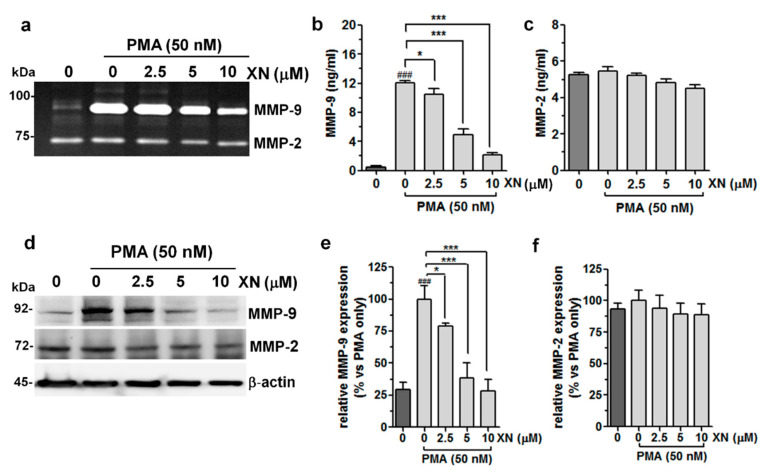
Effects of XN on the activity/expression of MMP-9 and MMP-2 in PMA-stimulated A549 cells. The cells were pre-incubated with XN for 4 h, followed by PMA co-stimulation for 24 h. (**a**) The gelatinolytic activity of MMP-9 and MMP-2 was assayed using gelatine zymography. The concentrations of MMP-9 (**b**) and MMP-2 (**c**) in the supernatants were determined with the ELISA assay. The results are the mean ± SD of three independent experiments, * *p* < 0.05, *** *p* < 0.001 in comparison to the PMA-only treatment; ### *p* < 0.001 in comparison to the non-stimulated cells; one-way ANOVA test. (**d**) The levels of MMP-9 and MMP-2 protein expression from whole cell lysates were measured with the Western blot analysis. β-actin was used as an internal control. The intensity of bands for MMP-9 (**e**) and MMP-2 (**f**) from the Western blot was quantified by densitometry analysis, where the untreated PMA-induced cells represented 100%. The results are the mean ± SD of three independent experiments, * *p* < 0.05, *** *p* < 0.001 in comparison to the PMA-only treatment, ### *p* < 0.001 in comparison to the non-stimulated cells; one-way ANOVA test.

It is well documented that MMPs, in particular MMP-2 and MMP-9, which digest type-IV collagen and ECM, are strongly associated with the metastatic potential of many types of tumour cells, and their over-expression confers a worse prognosis in the early stage of lung adenocarcinoma [30,31]. For this reason, we verified whether XN could modulate the PMA-stimulated enzymatic activity and/or expression of these proteins. The zymography assay (Figure 2a) demonstrated that the incubation with PMA strongly elevated MMP-9 activity, but only slightly increased MMP-2 activity.

The pre-treatment of the A549 cells with XN prevented the PMA-induced MMP-9 activity. The strongest inhibitory effect of XN was observed at 10 µM (Figure 2a). In turn, none of the XN concentrations applied, causing a considerable decline in MMP-2 activity. These results were compatible with those provided by the ELISA assays, showing an increase in the PMA-stimulated MMP-9 production and its significant reduction after incubation with XN (Figure 2b). The MMP-2 expression seemed to be unaffected in similar experimental conditions (Figure 2c). Moreover, the Western blot analysis supported the suppressive effect of XN on the expression of the MMP-9 protein (Figure 2d,e) by approx. 61% and 72% at the concentrations of 5 µM and 10 µM, respectively, in comparison to the cells subjected only to the PMA treatment. In contrast to MMP-9, the level of MMP-2 expression was not altered by XN (Figure 2d,f). These studies clearly indicate the potent specific activity of this compound against the PMA-induced MMP-9 expression.

### 3.3. XN Suppresses the Expression of TIMP-1

**Figure 3:**
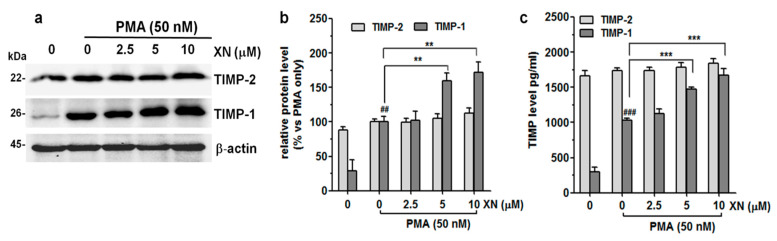
Effect of XN on the endogenous and exogenous production of TIMP-1 and TIMP-2 in PMA-induced A549 cells. (**a**) After the 4-h pre-treatment with XN followed by 24-h co-incubation with PMA, the levels of TIMP-1 and TIMP-2 were determined by immunoblotting. (**b**) The intensity of protein bands from Western blot was quantified by densitometry analysis, where the untreated PMA-induced cells represented 100%. The results are presented as the mean ± SD of three independent experiments, ** *p* < 0.01, in comparison to the PMA-only treatment, ## *p* < 0.01 in comparison to the non-stimulated cells; one-way ANOVA test. (**c**) Following the XN treatment, the supernatants from the A549 cell cultures were collected and the levels of TIMP-1 and TIMP-2 were assayed with ELISA. The results are the mean ± SD of three independent experiments, *** *p* < 0.001 in comparison to the PMA-only treatment, ### *p* < 0.001 in comparison to the non-stimulated cells; one-way ANOVA test.

It has been reported that MMPs can be negatively modulated by a specific endogenous tissue inhibitor of metalloproteinase proteins (TIMPs). The interaction of MMP-9, especially with TIMP-1 is critical for the inhibition of its proteolytic function [33]. To evaluate whether the altered expression/activity of MMP-9 could be, at least in part, related to the effect of XN on the expression of TIMP-1 and/or TIMP-2, the levels of these molecules in cell lysates and supernatants from the XN-treated or non-treated PMA-stimulated cells were estimated using the Western blot and ELISA assays, respectively. As shown in Figure 3a,b, XN applied in all concentrations resulted in only minor changes in the expression levels of the TIMP-2 protein in the PMA-stimulated A549 cells. In contrast, the TIMP-1 protein expression was markedly elevated in the presence of 5 µM and 10 µM of this compound. At its highest concentration, the level of TIMP-1 expression was almost 75% higher than in the PMA-only treated group (Figure 3a,b).

Moreover, XN (except 2.5 µM) also significantly induced the secretion of the TIMP-1 protein, but slightly stimulated the release of TIMP-2 (Figure 3c). Compared with the PMA-stimulated XN-untreated cells, the levels of TIMP-1 and TIMP-2 increased from 1033 ± 31.6 pg/mL to 1668 ± 97.8 pg/mL (*p* < 0.001) and from 1738 ± 34.7 pg/mL to 1845 ± 60.6 pg/mL (*p* > 0.05), respectively, in response to 10 µM of XN. These experiments indicate that the reduced level of MMP-9 activity, as indicated in the previous study, was partly the result of XN-mediated TIMP-1 upregulation.

### 3.4. XN Suppresses the Release of VEGF and TGFβ from PMA-Stimulated A549 Cells

**Figure 4:**
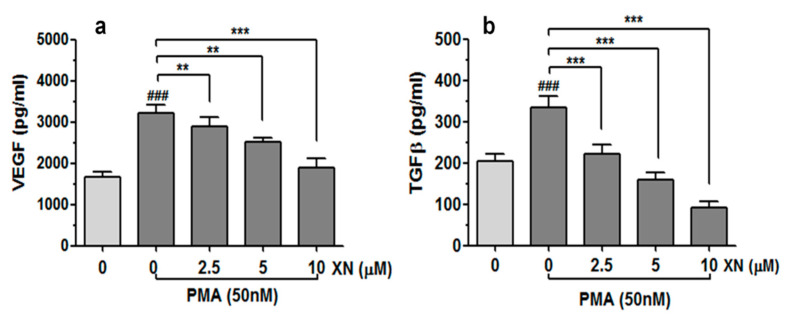
Effect of XN on VEGF and TGF-β release from PMA-stimulated cells. The A549 cells were pre-incubated with the indicated concentrations of XN for 4 h and then co-incubated in the presence of PMA for 24 h. Following the treatment, the concentration of VEGF (**a**) and TGF-β (**b**) in the supernatants was assessed using the ELISA assay. The results are presented as the mean ± SD of three independent experiments, ** *p* < 0.01, *** *p* < 0.001 in comparison to the PMA-only treatment, ### *p* < 0.001 in comparison to the non-stimulated cells; one-way ANOVA test.

In order to examine whether XN is able to interfere with the PMA-induced expression of the key regulators of angiogenesis and tumour aggressiveness, the levels of VEGF and TGF-β proteins in the A549 culture media were quantified with the ELISA method, as described in the Material and Methods section. As shown in Figure 4a,b, the treatment of XN significantly reduced the PMA-induced production of both analysed factors in the studied cancer cells in a concentration-dependent manner. Compared to the PMA-stimulated XN-untreated cells, the amounts of VEGF and TGF-β released from the cells decreased by approx. 1.6-fold and 3.7-fold when the highest XN concentration was applied (*p* < 0.001).

### 3.5. XN Influences EMT-Related Phenotype Markers in PMA-Stimulated Cells

**Figure 5:**
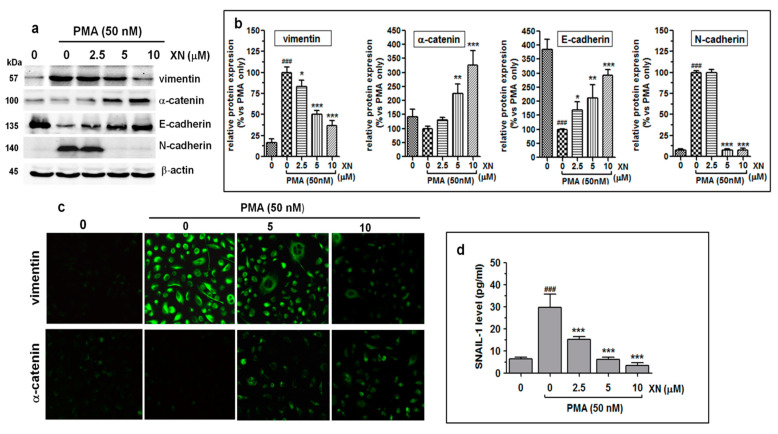
XN inhibits the progress of epithelial-mesenchymal transition in PMA-induced A549 cells. (**a**) The A549 cells were pre-treated with XN at the indicated concentrations for 4 h prior to 24-h co-stimulation with PMA (50nM). Next, they were subjected to Western blotting to analyse the levels of vimentin, α-catenin, E-cadherin, and N-cadherin proteins. β-actin was used as an internal control. (**b**) The intensity of protein bands from immunobloting was quantified by densitometry analysis, where the untreated PMA-induced cells represented 100%. The results are the mean ± SD of three independent experiments, * *p* < 0.05, ** *p* < 0.01, *** *p* < 0.001 compared to the PMA-only treatment, ### *p* < 0.001 in comparison to the non-stimulated cells; one-way ANOVA test. (**c**) After the treatment with the selected concentrations of XN followed by PMA stimulation, the cells were incubated with anti-vimentin antibody or anti-α-catenin antibody followed by FITC labelled secondary antibody. The subcellular identification of vimentin and α-catenin was performed under a fluorescence microscope at 200 x magnification. (**d**) XN decreases the expression of the Snail-1 transcription factor. After the treatment, the amounts of the Snail-1 protein in the total cell extracts were assessed with the ELISA assay. The results are the mean ± SD of three independent experiments. *** *p* < 0.001 compared to the PMA-only treatment, ### *p* < 0.001 in comparison to the non-stimulated cells; one-way ANOVA.

Since the increased motility and invasion of tumour cells is usually preceded by epithelial-mesenchymal transition (EMT), i.e., a master event in tumour metastasis, we determined the effect of XN on the expression of E-cadherin, N-cadherin, α-E-catenin, vimentin, and Snail-1, which are the critical regulators of this phenomenon. The immunoblot results demonstrated that the 4-h pre-incubation of the PMA-activated cells with XN led to marked concentration-dependent changes in the expression of both the epithelial markers (E-cadherin and α-catenin) and the mesenchymal vimentin (Figure 5a).

As revealed by the densitometric analysis (Figure 5b), the relative levels of E-cadherin and α-catenin expression at 10 µM of XN were approx. 192% and 226% higher than those in the PMA-only treated cells, respectively. In turn, in the same treatment conditions, the amount of the vimentin protein was reduced by 64%, compared to the PMA-alone treatment (Figure 5b). XN at a concentration of 2.5 µM did not have any effect on the level of N-cadherin. In contrast, at the concentrations of 5 µM and 10 µM, a rapid (almost 93%) decrease in its inducible expression was observed (Figure 5a,b).

In addition, the α-catenin upregulation and simultaneous vimentin downregulation in the XN-treated and PMA-stimulated cells were also indicated using immunofluorescence staining (Figure 5c). Similar to the Western blot assay, the greatest alterations in the expression of the ETM contributors were detected in the presence of 10 µM of XN. Parallel to these observations, we also found the reduced expression of the Snail-1 protein, i.e., a transcription factor, to be functionally associated with the regulation of EMT genes. As shown in Figure 5d, already the low concentration of XN (2.5 µM) significantly (almost 2-fold, *p* *<* 0.001) abolished the PMA-induced Snail-1 protein expression. An even stronger effect was triggered by the concentrations of 5 µM or 10 µM XN, decreasing the Snail-1 expression level over 4.7-fold or 8.5-fold (compared to the PMA-stimulated XN-untreated cells), respectively. These data indicate that the negative regulation of the EMT phenotype of the A549 cells by XN was probably attributed to the downregulation of the Snail-1 protein. Thus, the ability of XN to promote mesenchymal-epithelial transition may be responsible for the inhibition of the migration and invasion of the A549 cells.

### 3.6. XN Decreases the PMA-Induced Phosphorylation of FAK, AKT, and ERK/2 Kinases in A549 Cells

**Figure 6:**
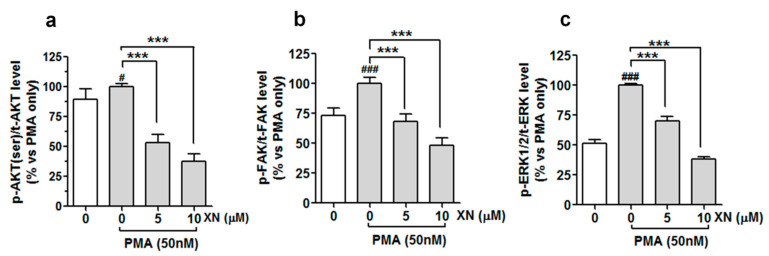
XN interferes with PMA-dependent activation of AKT, FAK, and ERK1/2, kinases in A549 cells. The cells were pre-treated with XN for 4 h and then co-treated with PMA for 24 h; the amounts of phosphorylated ERK1/2, FAK, and AKT were determined with the ELISA assay. Quantitative analysis of (**a**) p-AKT/tAKT, (**b**) p-FAK/tFAK, and (**c**) p-ERK/tERK ratio. Data are expressed as means ± SD of three independent experiments. *** *p* < 0.001 compared to the PMA-only treatment, # *p* < 0.05 or ### *p* < 0.001 compared to the non-stimulated cells; one-way ANOVA test.

Integrin/FAK, PI3/AKT, and MAPK signalling pathways have been reported to be involved in the activation of the EMT program and induction of several molecular markers responsible for the metastatic potential of tumour cells [47]. Therefore, we investigated the pattern of the expression of selected signalling molecules of these pathways to explain the molecular mechanism underpinning the XN-mediated inhibition of metastatic progression in PMA-activated cells. The results of the quantitative ELISA assay (Figure 6a–c) demonstrated that cells cultured in the presence of PMA (50 nM) displayed a noticeable increase in the phosphorylation levels of AKT (ser473), FAK (Y-397), and ERK1/2 (Thr202/Tyr204) kinases, in comparison to the vehicle non-stimulated cells.

**Figure 7:**
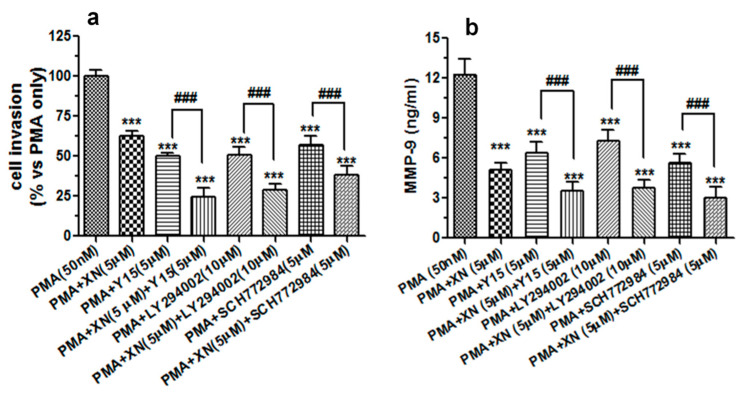
The XN-mediated suppression of the PMA-induced invasive potential of A549 cells and MMP-9 production is associated with alteration of FAK/AKT and MAPK/ERK signalling pathways. The A549 cells were exposed to a single treatment (5 µM XN, LY294002, Y15, or SC772984) or a combination treatment (XN + Ly294002, XN + Y15, or XN + SC772984) for 4 h, and then incubated in the presence of PMA (50 nM) for 24 h. (**a**) The invasive ability of the treated cells was studied using the Transwell in vitro invasion assay. The invasion of the untreated PMA-stimulated cells was established as 100%. *** *p* < 0.001 compared to the PMA-stimulated cells; ### *p* < 0.001; one-way ANOVA test. (**b**) After the single or combination treatment (as indicated above), the supernatants from the PMA-stimulated cells were subjected to the ELISA assay to analyse the levels of the MMP-9 protein. The results are the mean ±SD from three independent experiments. *** *p* < 0.001 compared to the PMA-treated cells, ### *p* < 0.001; one-way ANOVA test.

This suggests a stimulating impact of the PMA on the activation of all the kinases tested. However, following the treatment with 5 µM of XN prior to the co-incubation with PMA, the level of phospho-AKT, phospho-FAK, and phospho-ERK1/2 declined to almost 53%, 68%, and 70% and to 37%, 48%, 38% of the PMA-alone treated group after the 10 µM treatment, respectively (Figure 6a–c). A similar downward trend in the inducible phosphorylation of FAK, AKT, and ERK1/2 was also obtained by the pre-treatment of the cells with the pharmacological inhibitor of FAK kinase (Y15, 5 µM), ERK1/2 kinase (SCH772984, 5 µM), and PI3/AKT kinase (LY294002, 10 µM), respectively (data not shown). To validate the importance of these signalling molecules in the molecular events responsible for the anti-metastatic potential of XN, the A549 cells (before the 24-h PMA stimulation) were pre-treated with Y15 (5 µM), SCH772984 (5 µM), and LY294002 (10 µM) alone or in combination with XN (5 µM). Next, the cell invasion assay and the MMP-9 ELISA assay were performed. The examination of cell invasiveness after the treatments revealed that the specific inhibitors used in this experiment were capable of reducing the PMA-induced cell invasion, and their effects were slightly stronger than that of XN (Figure 7a).

Interestingly, the combination treatment with each inhibitor potentiated the anti-invasive activity of XN. Moreover, the exposure of the cell cultures to Y15, SCH772984, or LY294002 also remarkably decreased the amounts of MMP-9 secreted from the PMA-stimulated cells. In addition, these inhibitors intensified the XN-mediated MMP-9 suppression (Figure 7b). These results may thus imply that the anti-migrastatic potential of XN is at least in part accompanied by the inhibition of the PMA-associated stimulation of ERK 1/2 and FAK/AKT signalling in A549 cells.

## 4. Discussion

The emergence of the invasive potential of the tumour is associated with the acquisition of various genetic and/or epigenetic alterations that allow cancer cells to spread and form local and distal metastases. Since tumour metastasis and recurrence are the major impediments to clinical success in the treatment of lung cancer patients, novel targeted therapeutic agents with high efficacy and low systemic toxicity are urgently required to hinder metastatic processes [23]. The available data indicate a beneficial multidirectional impact of XN on the human health and highlight its protective function against a wide range of solid tumours [20]. Moreover, there is some evidence that XN may be useful for treating non-small cell lung cancer. A recent study conducted by Long et al. [48] has suggested a role of this compound in potentiating the anticancer effect of cisplatin on the metastatic non-small cell lung cancer H1299 cell line. In addition, our earlier experiments on the A549 human lung cancer cell line reported the anti-proliferative and pro-apoptotic properties of XN [11]. More importantly, the latest research reported by Gao et al. [12] confirmed the anti-NSCLC effect of XN, not only in a cell culture system but also in a xenograft mouse model, indicating the importance of downregulation of ERK1/2 activation, as well as Fra1 and cyclin D1 expression in this phenomenon. Whether other cellular mechanisms may be implicated in the XN-mediated lung tumour suppression remains to be addressed. In this paper, we provided the first data on the anti-invasive potential of XN in relation to the human lung adenocarcinoma A549 cell line. The present study showed that XN suppressed the PMA-mediated invasive phenotype of A549 cells by targeting multiple protein factors (i.e., MMP-9, VEGF, TGF-β, TIMP-1, E-cadherin, vimentin, N-cadherin and Snail-1) crucial for extracellular matrix (ECM) remodelling, epithelial-mesenchymal transition, cell migration, and angiogenesis. All these XN effects were likely to be associated with the inhibition of the PMA-mediated activation of ERK1/2, AKT, and FAK signalling molecules.

Metastatic progression is a precisely controlled multi-step biological process involving specific cell-to-cell and cell-to-ECM interactions, cell movement, and invasion. Hence, the inhibition of one or more of these interrelated events is a rational strategy for metastatic disease therapy. Several published studies on breast and prostate cancer have previously documented the ability of XN to block tumour progression by decreasing the migratory and metastatic potential of tumour cells [49]. The results of our study revealed that XN in non-cytotoxic concentrations markedly reduced the migration rate and invasiveness of A549 cells, confirming the anti-invasive mode of XN action. To elucidate how XN can trigger these effects, we assessed its influence on the expression or secretion of various factors allowing malignant cells to penetrate surrounding and distant tissues.

MMPs particularly contribute to tumour invasion through an influence on the collective migration of cancer cells. MMP-2 and MMP-9 were identified as factors associated with the metastatic phenotype of lung adenocarcinoma cells [31,32]. Hence, the downregulation of these enzymes may be crucial in inhibiting the protease-dependent invasion of tumour cells and the regulation of metastatic cascades. In the present study, we found that XN affected the PMA-mediated gelatinolytic activity of the conditioned medium of A549 cells. Surprisingly, of the two MMPs tested, only MMP-9 was strongly susceptible to the inhibitory effect of the compound. The XN specificity against MMP-9 was also confirmed in the ELISA and Western blot experiments. Since the activity of MMPs is tightly controlled by TIMPs, we assumed that alteration in MMP-9 activation might be in part related to TIMP-1 and/or TIMP-2 overproduction. As evidenced, there was a great increase in the TIMP-1 protein level in response to the different XN concentrations. These results imply a possible role of XN in the TIMP-1-dependent regulation of MMP-9 activity, as TIMP-1 forming a specific complex with pro-MMP-9 blocks its activation. The stimulation of endogenous TIMP-1 production, along with the inhibition of MMP-9 expression, may be one of the suggestive reasons why the studied cells stopped to transverse through the undigested matrigel in the invasion assay. The diverse and cell-type specific effects of XN on the expression of MMPs and TIMPs were previously described by other authors. Yamaguchi et al. [50] suggested anticollagenase activities of this prenylflavonoid against MMP-1 and MMP-8. Dell’Eva et al. [51] demonstrated the reduced release of both MMP-9 and MMP-2 from XN-treated leukemic cell lines. Moreover, in TPA-induced HT-1080 fibrosarcoma cells, XN suppressed both the activation and expression of MMP-9 and MMP-2, reduced the MT1MMP mRNA level, and induced a substantial TIMP-1 and TIMP-2 depletion [52].

A successful anti-metastatic lung cancer therapy requires drugs that can interfere with angiogenesis. According to the recent literature reports, XN seems to be a strong angiogenesis suppressor [19,52,53,54]. In our more recent study on multiple myeloma cells [13] and those conducted by Dell’Eva et al. [51] on leukemic cells, this compound was found to attenuate the expression of VEGF, which is the most prominent proangiogenic growth factor. Similarly, in the PMA-stimulated A549 cell line, a significant inhibition of VEGF release to the culture media was demonstrated in the presence of various concentrations of XN. Since VEGF is implicated in the malignant transformation and angiogenic behaviour of lung cancer cells and confers drug resistance, anti-VEGF therapy with XN might be a realistic option to prevent lung cancer cells from invading and metastasize and to improve the efficacy of the standard chemotherapy.

TGF-β is regarded as another paramount molecule involved in cancer advancement through various mechanisms. As mentioned in the Introduction, TGF-β has been demonstrated to evoke pathological EMT, i.e., a complex process inseparably associated with temporary loss of epithelial hallmarks and the acquisition of a highly motile mesenchymal phenotype necessary for aggressive tumour potential and malignant expansion [28,55]. More importantly, the experimental blockade of TGF-β and its downstream signalling components has been found to be sufficient to inhibit the EMT incidence and thereby cancer invasion [47]. In the light of these data and those showing a strong correlation between elevated TGF-β1 levels in serum and/or non-small lung cancer tissues and advanced stage malignancy [47], it was worth assessing how XN works in relation to TGF-β and its regulated EMT proteins, such as E-cadherin, α-catenin (representative epithelial markers), N-cadherin, and vimentin (major mesenchymal markers). It is noteworthy that E-cadherin and α-E-catenin are both frequently depleted in all histological types of primary lung cancers [26,27]. Many investigations have recently reported that the disruption of the E-cadherin complex and/or the functional loss of any of its components is responsible for the cell–cell contact weakness, rapid proliferation, increased cellular movement, and malignant progression [56]. In contrast to the epithelial indicators, vimentin has been described to be strongly overexpressed in highly metastatic lung cancer cell lines and in patients with advanced NSCLC disease [25,29]. Additionally, the elevated expression of N-cadherin was associated with the aggressive phenotype and inferior patient prognosis in lung cancer [57].

In the present study, we showed that the basal TGF-β expression level in the A549 cells was significantly increased by PMA, implying that TGF-β signals could enhance PMA-activated EMT in A549 cells. Notably, the PMA-mediated TGF-β production was successfully reduced by the XN treatment, which consistently led to an increase in the α-catenin and E-cadherin levels, but decreased the vimentin and N-cadherin expression. Moreover, the alterations in the levels of both TGF-β and EMT markers were well correlated with the anti-metastatic characteristic of this prenylflavonoid. Therefore, the downregulation of TGF-β followed by molecular and phenotypic switching retardation may play a vital role in the XN-mediated inhibition of malignant transformation of NSCLC cells and their invasiveness. Nevertheless, detailed studies are needed to support our conclusion.

It is widely accepted that Snail-1 is central to the mechanism of EMT and cancer progression [58]. Liu et al. [59] has recently revealed that Snail-1 overexpressing lung cancer cell lines was more motile and tumorigenic than those without endogenic Snail-1 expression and showed remarkably lower responsiveness to cisplatin treatment. Due to the clinical relevance of Snail-1 in lung cancer cell dissemination and NSCLC disease prognosis, the XN impact on its production was further estimated. Here, we found that XN diminished the content of the Snail-1 protein in the PMA-activated A549 cell line in a concentration-dependent manner. Our study provided the first evidence that XN was able to interfere with the EMT-related process by targeting molecular events at/or upstream of Snail-1. The anti-EMT mode of the XN action has already been postulated by a few research groups. More recently, Kimawaha et al. [60] demonstrated in a cholangiocarcinoma (CCA)-induced hamster animal model that XN (alone or in combination with anthelmintic drug praziquantel) prevented opisthorchiasis-associated cholangiocarcinoma development by attenuating the expression of Twist-1 (EMT-related transcription factor). Moreover, Viola et al. [61] reported that the downregulation of EMT molecules such as MCL2, S100A4, and paxillin as a consequence of NF-κB inactivation was linked to an anti-invasive effect of XN on human breast cancer cells. All these facts confirm that XN has potent anti-EMT properties, which allow the effective blunting of the invasive and metastatic phenotype of epithelial origin malignant tumours and prevented EMT-driven drug resistance.

In many experimental in vitro and/or in vivo models, XN was reported to inhibit tumour angiogenesis/invasion and tumour growth, mainly through the modulation of PI3K/AKT and mitogen-activated protein kinase (MAPK) signalling and their downstream substrates [20,62]. Both AKT and ERK are intracellular signalling molecules aberrantly expressed in NSCLC tissues, and their activation can stimulate angiogenesis and tumour metastasis [39,40]. Our previous work documented that XN was capable of targeting the MEK/ERK/CREB MAPK signalling axis, which probably slowed down the cell cycle progression and mitotic potential of non-stimulated A549 cells [11]. In the present study, XN abolished the inducible ERK phosphorylation and additionally abrogated the stimulating effect of PMA on AKT activation, which suggested that both ERK/MAPK and PI3/AKT pathways might be involved in the XN-driven regulation of PMA-mediated invasion of A549 cells. This finding was supported by the fact that treatment with SC772984 (a specific inhibitor of ERK1/2 kinase) or LY294002 (a blocker of PI3 kinase) resulted in a noticeable decrease in the number of invasive cells and secreted MMP-9 and, importantly, the simultaneous incubation of XN with SC772984 or LY294002 potentiated the inhibitory effects of these agents. Furthermore, consistent with earlier data [49], XN was revealed to impair the phospho-FAK expression, accompanied by a reduction in PMA-mediated A549 cell invasion and MMP-9 production. It has been established that FAK is strongly implicated in lung cancer progression and metastasis. Enhanced FAK phosphorylation was reported to activate ERK and PI3, contributing to improvement of the migratory and invasive ability of cancer cells [41,42]. Moreover, Woo et al. [63] showed that inactivation of FAK by another natural agent-daurinol was associated with attenuated expression of MMP-9, MMP-2, and uPA in the A549 and MDMB-231 cell lines and with limited lung cancer metastasis in vivo. Accordingly, in our experiment, blocking FAK signalling with Y15 (a specific FAK inhibitor) or XN disturbed the PMA-mediated invasion and MMP-9 production in the A549 cells. More importantly, the co-treatment with XN and Y15 made the cells even more sensitive to the anti-invasive and anti-MMP-9 action of XN. Therefore, it is possible that inhibition of FAK phosphorylation potentiates the inhibitory effect of XN on the PMA-induced aggressive phenotype of A549 cells.

## 5. Conclusions

This is the first report indicating the ability of XN to alleviate the PMA-induced invasive activity of A549 cells by decreasing ERK1/2 signalling and the downregulation of the PI3/AKT and FAK signal cascade. The suppressive effect of XN on all these protein kinases possibly disturbed invasion-related molecular events to finally impair the migration/invasion ability of the studied cells. Thus, the results suggest a possibility that XN may be a promising compound in delaying or attenuating the transformation of lung cancer cells into a more aggressive/invasive phenotype. However, further investigations in an in vivo lung tumour model are urgently needed to verify the anti-invasive properties of XN against this cancer.

## Data Availability

Not applicable.

## Supplementary Materials

The following are available online at https://www.mdpi.com/article/10.3390/cells10061484/s1, Figure S1: Effect of XN on induction of apoptosis in PMA-stimulated A549 cells. Figure S2: Effect of XN on proliferation of the PMA-stimulated lung cancer cell line.
