# Supplementary material for: Xanthohumol Impairs the PMA-Driven Invasive Behaviour of Lung Cancer Cell Line A549 and Exerts Anti-EMT Action
Source: Cells. 2021 Jun 12;10(6):1484. doi: 10.3390/cells10061484 (PMC8231538; doi:10.3390/cells10061484)
Supplement: Supplementary file 1 [file cells-10-01484-s001.zip › cells-1216415-supplementary.pdf]

Xanthohumol impairs the PMA-driven invasive behaviour of lung cancer cell line A549 and exerts anti-EMT action.

Adrianna Sławińska-Brych [1,*], Magdalena Mizerska-Kowalska [2], Sylwia Katarzyna Król [3], Andrzej Stepulak [4] and Barbara Zdzisińska [2]

Supplementary Methods

1. Assesement of caspase-3 activity

For caspase activity assessment, the cells were plated in 6-well tissue culture plates (4 × 10^5 cells/well). Following 24 h of treatment with a combination of PMA (50nM) and XN (0-40 µM), the cells were labelled with PE-conjugated anti-active caspase-3 antibody (BD Biosciences, BD Pharmingen™, USA) according to the manufacturer's instructions and analyzed with a FACSCalibur flow cytometer using CellQuest software.

2. Assesement of cell proliferation

The cells (1 × 10^4 cells/well) in a complete medium were seeded into 96-well plates (100 µl/well) and then exposed to a combination of PMA and XN (0-80 µM,) for 24 h. Following the treatment, cell proliferation was estimated by measuring the amount of BrdU incorporated into nuclear DNA using a commercial Cell Proliferation ELISA, BrdU Kit (Roche Molecular Biochemicals, Germany) according to the manufacturer's instructions. The absorbance values were measured at 450 nm using an ELISA reader

Supplementary Figure 1.

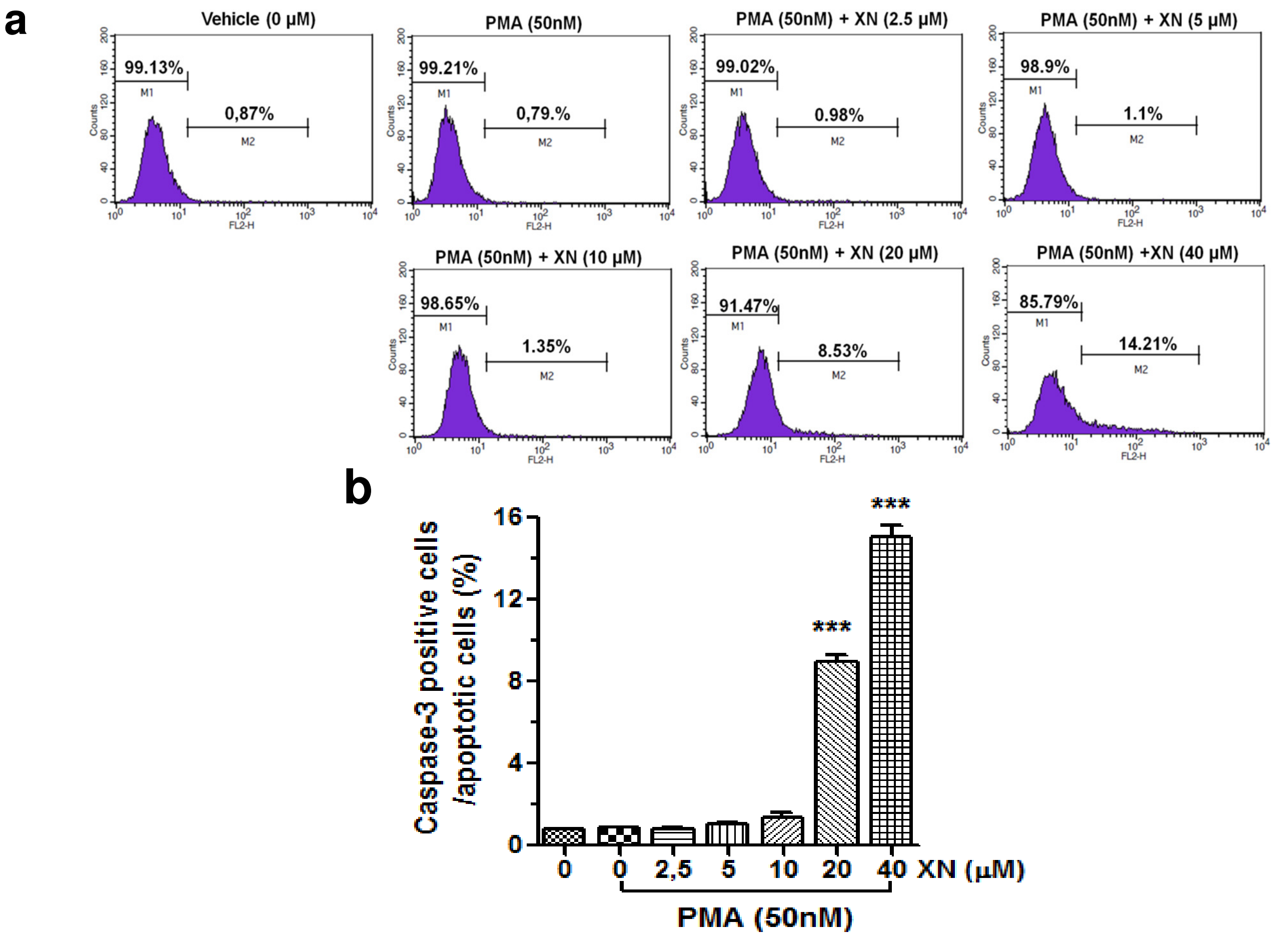

Figure S1. Effect of XN on induction of apoptosis in PMA-stimulated A549 cells. After the 24-h exposure to PMA (50 nM) alone or in combination with increasing concentrations of XN, the cells were subjected to flow cytometry

analysis of active caspase-3. (**a**) Representative histograms of the A549 cell line. Symbols M1 and M2 represent peaks for viable (caspase-3 negative cells) and apoptotic cell fractions (caspase-3 positive cells), respectively. **(b)** Quantification of caspase-3 activity in the A549 cells. The results are the mean ± SD from three independent experiments (***p < 0.001 in comparison to the PMA only-treated cells; one-way ANOVA test).

Supplementary Figure 2.

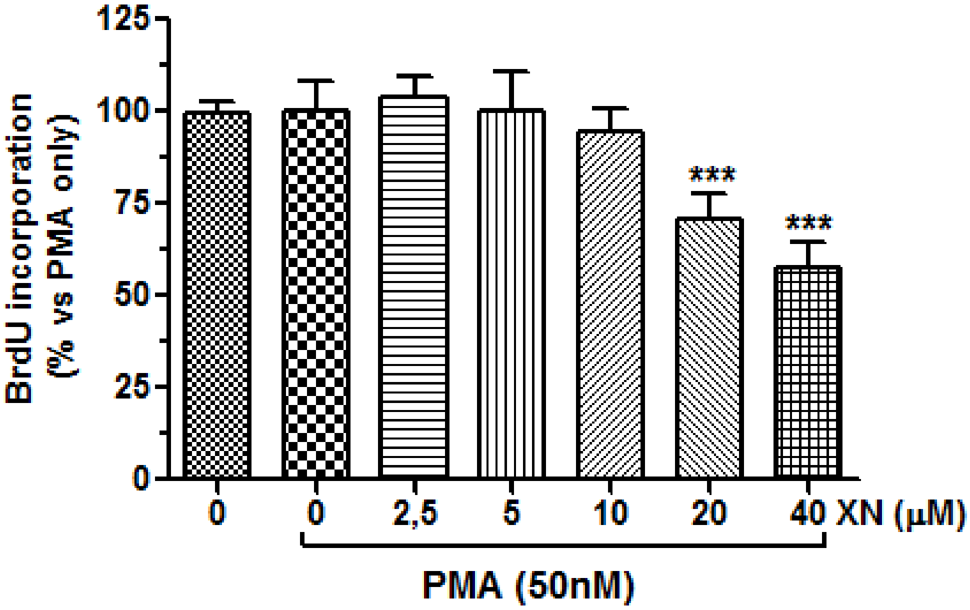

Figure S2. Effect of XN on proliferation of the PMA-stimulated lung cancer cell line. A549 cells were cultured alone or exposed to combinations of 50 nM PMA with increasing concentrations of XN (0-80 μM) for 24 h. After the treatment, the proliferation of the A549 cells was determined with the BrdU assay. The results represent the mean ± SD of three independent experiments; statistical significance at p < 0.001 *** in comparison to the PMA treated cells; one-way ANOVA test.
